# Degradation and Failure Phenomena at the Dentin Bonding Interface

**DOI:** 10.3390/biomedicines11051256

**Published:** 2023-04-23

**Authors:** Lamia Sami Mokeem, Isadora Martini Garcia, Mary Anne Melo

**Affiliations:** 1 Ph.D. Program in Biomedical Sciences, University of Maryland School of Dentistry, Baltimore, MD 21201, USA; 2 Division of Operative Dentistry, Department of General Dentistry, University of Maryland School of Dentistry, Baltimore, MD 21201, USA

**Keywords:** dental restoration failure, smear layer, dental etching, collagen degrading enzymes

## Abstract

Damage in the bonding interface is a significant factor that leads to premature failure of dental bonded restorations. The imperfectly bonded dentin-adhesive interface is susceptible to hydrolytic degradation and bacterial and enzyme attack, severely jeopardizing restorations’ longevity. Developing caries around previously made restorations, also called “recurrent or secondary caries,” is a significant health problem. The replacement of restorations is the most prevailing treatment in dental clinics, leading to the so-called “tooth death spiral”. In other words, every time a restoration is replaced, more tooth tissue is removed, increasing the size of the restorations until the tooth is eventually lost. This process leads to high financial costs and detriment to patients’ quality of life. Since the complexity of the oral cavity makes prevention a challenging task, novel strategies in Dental Materials and Operative fields are required. This article briefly overviews the physiological dentin substrate, features of dentin bonding, challenges and clinical relevance. We discussed the anatomy of the dental bonding interface, aspects of the degradation at the resin-dentin interface, extrinsic and intrinsic factors affecting dental bonding longevity, perspectives on resin and collagen degradation and how these subjects are connected. In this narrative review, we also outlined the recent progress in overcoming dental bonding challenges through bioinspiration, nanotechnology and advanced techniques to reduce degradation and improve dental bonding longevity.

## 1. Introduction

Despite the remarkable advances in adhesive systems, the dentin-adhesive interface remains the “Achilles’ heel” of composite restorations [1]. The exposure of dentin-adhesive interface to the oral cavity can lead to poor marginal adaptation, marginal discoloration, and bacterial penetration [2]. These events may contribute to the development of secondary caries and retention loss of the resin restoration [2]. Replacement of failed restorations accounts for almost 70% of all restorative dentistry in the United States [3]. This unfavorable outcome has increased patient costs in time and money [3].

Over the years, with more and more bonded restorations using resin-based materials and advances in dental adhesives’ formulation and techniques, the adhesion to dentin still challenges the clinical outcomes expected of these materials. The dentin substrate is significantly linked to bonding performance and degradation, which is related to the singular characteristics of this complex tissue—first, the hydrated structural heterogeneity and composition variation with locations offering particular structure-property relationships. Furthermore, dentin is modified by physiological, aging, and disease processes to create different types of dentin substrates. This variability increases the likelihood of a resin-dentin bond’s degradation and failure to occur [4].

To bond to dentin, a series of topical treatments are applied to dentin to change its hydrophilic, crystalline, relatively impermeable acid-labile surface into a more hydrophobic, organic, highly porous surface and acid-resistant surface [5]. Hence, a micromechanical interlocking bonding can be achieved, which involves the infiltration and subsequent entanglement of adhesive resin into the dentin collagen matrix to form the hybrid layer [6]. Unfortunately, adhesives cannot infiltrate 100% into the collagen fibril network of the demineralized dentin, exposed by acid-etching or self-etch processes [7,8]. As a result of incomplete resin infiltration into the demineralized dentin, the collagen matrix will be exposed and susceptible to hydrolysis, causing a progressive failure [2,9].

Hybrid layer degradation at the dentin-adhesive interface is believed to be the prime reason for failure [10]. This results from a combinatorial effect of various mechanical and chemical factors, including the hydrolysis and enzymatic degradation of the exposed collagen and the adhesive resin [10]. In collagen fibrils, degradation could start immediately after applying the bonding agent or after the adhesive material polymerization [7,8]. According to Hashimoto and others [11,12], the first involves the activation of enzymes that slowly degrade collagen fibrils exposed by acid etching, known as matrix metalloproteinases (MMPs). The latter degradation is initiated from the hydrolytically susceptible groups in the molecular structure of methacrylate-based resin monomers, such as ester, urethane, hydroxyl, carboxyl and phosphate.

Although an increasing number of studies have made some breakthroughs in understanding the dentin bonding interface mechanism, many barriers must be overcome. Long-term bonding performance and dentin biostability, the inherent challenge related to the dentin as a substrate, harsh oral environmental conditions (biofilms, saliva, fatigue, etc.) and the current adhesive chemical characteristics remain the major obstacles to reaching the long-term service inside the mouth expected from bonded restorations. Hence, this article briefly overviews the physiological dentin substrate, features of dentin bonding, challenges and clinical relevance. In addition, we outline the recent progress in overcoming these challenges through bioinspiration, nanotechnology and advanced techniques.

## 2. The Anatomy of Dental Bonding Interface

### 2.1. Dentin Bonding

**Figure 1:**
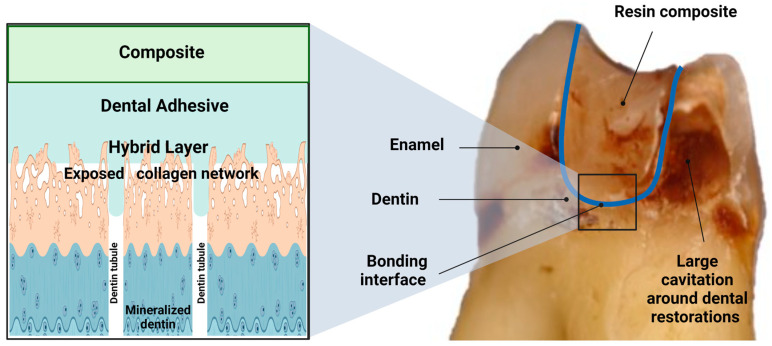
A schematic illustration to show a defective bonded restoration (**right**). The image displays a restored tooth’s main components and illustrates secondary caries around the restoration. The blue line represents the bonding interface composite/tooth. At a greater zoom (**left**), the typical components of the dentin bonding interface are described.

In dentin, around 50% of its volume is inorganic, while 30% is organic and 20% is water [13]. Its mineral content is enriched by carbonate and calcium apatite [14]. Its organic component is mainly collagen type I, with a minor content of proteins such as phosphoproteins, glycoproteins and carboxyglutamate-containing proteins [15]. A particular characteristic of dentin is that the dentinal tubules traverse the structure from the pulp cavity to just below the dentin-enamel junction (DEJ) or dentin-cementum junction (DCJ). These tubules create tunnels between the pulp chamber’s odontoblastic cells and the respective links, as shown in Figure 1. Their density differs by location. It is at its lowest at the DEJ and highest near the pulp chamber. Dentinal tubules are surrounded by a highly mineralized peritubular dentin composed of carbonate apatite [16]. The in-between tissue tubules are known as intertubular dentin, and are composed of perpendicular unmineralized collagen fibrils [17]. Dentin composition is affected by its position within the tooth, age and the presence or absence of the disease [18], and understanding this composition is essential in improving the bonding [19].

### 2.2. Smear Layer

The smear layer is inorganic debris resulting from frictional heat and plastic/elastic deformation during tooth preparation that is 0.5 to 2 μm thick [16]. It covers the intertubular dentin and penetrates several microns into the tubules. This layer should be removed or dissolved to expose the tubules to achieve mechanical interlocking in the dentin bonding [17]. In 1980, Fusayama et al. reported that dentin etching with phosphoric acid would open the dentinal tubules, causing micromechanical adhesion and forming the hybrid layer [20].

### 2.3. Hybrid Layer

The hybrid layer is a three-dimensional polymer/collagen network that provides a continuous and stable bond between the adhesive and dentin [21,22]. Unfortunately, this ideal objective still needs to be attained. Instead, the hybrid layer has been described as the weakest bond in the adhesive-dentin interface [23,24]. During acid etching, the mineral phase is extracted from the dentin [25], creating a demineralized dentin composed of 30% collagen and 70% water by volume, causing the collagen fibers to be suspended in the water [26]. With air drying, the collagen will collapse, inhibiting the resin penetration by creating a barrier between the demineralized layer and underlying intact dentin, severely compromising the adhesive-dentin interface [27]. The hybrid layer’s clinical longevity depends on the concurrent action of many influencing factors. Physical and chemical aspects, occlusal chewing forces and the repetitive expansion and contraction stress due to temperature changes within the oral cavity are examples of these influencing factors [28,29,30]. On the other hand, chemical agents in dentinal fluid, saliva, food and beverages, in addition to the bacterial acid attack at the tooth/biomaterials interface, contribute to various patterns of degradation of unprotected collagen fibrils [31,32,33], elution of resin monomers and degradation of resin components [21,31,34,35,36,37].

### 2.4. Summary of Current Classification

**Figure 2:**
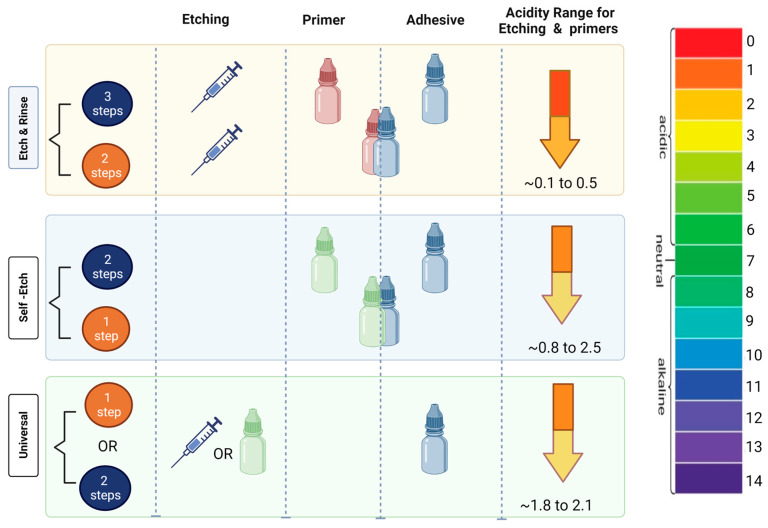
Classification of dental adhesive systems based on their interaction with dentin substrate according to Van Meerbeek et al. (**Top**) The etch & rinse adhesives require the etching step. The primer and adhesive can be presented in separate bottles (3 steps) or combined in one bottle (2 steps). (**Middle**) The self-etch adhesives do not require the etching step with phosphoric acid since the acidic functional monomers present in the composition of these adhesives will treat the smear layer and provide micro-surface retention. The etching efficacy of functional monomers is commonly determined in terms of pH. The right column represents the acidity of the adhesives. The colors from yellow to red indicate a range of pH acidic solutions. Darker colors represent lower pH in the range of 1–3. (**Bottom**) Universal adhesives, the latest development in the field. This class of adhesives can be used as self-etch, etch-and-rinse or enamel selective-etch. The universal adhesives contain monomer blends of mild to moderate acidity. Note that greater acidity may contribute to the degradation process of the denting bonding interface.

The dentin bonding systems have three main components: etchant, primer and bonding agent. Each component’s role is summarized in Table 1 [17,38,39]. Adhesive systems are classified according to how they interact with the dental substrate into (1) etch and rinse (E&R) and (2) self-etch (SE), according to Van Meerbeek et al. [40] and summarized in Figure 2.

In the E&R strategy, the acid etchant removes the smear layer and creates a superficial layer of demineralized dentin. To achieve a stable bond, water must be replaced entirely by adhesive in an exposed collagen mineral-free network [7,40,41]. Unfortunately, complete monomer infiltration into wet and demineralized dentin is not always succeeded, and incomplete infiltrated zones form at the bottom of the hybrid layer with collagen fibrils surrounded by water [42]. This phenomenon indicates that the top half of the hybrid layer contains a completed resin encapsulation of acid-etched collagen fibrils compared to the bottom half [43].

On the other hand, the SE strategy depends on the adhesive co-monomers in demineralizing and infiltrating the dentinal substrate to create a more homogenous resin infiltration of demineralized collagen fibrils compared to E&R systems [43,44,45]. However, the SE adhesion stability depends on the coupling efficiency between collagen fibrils and co-monomers. Therefore, despite differences in etching systems, priming and bonding are fundamental steps for adhesion, where they can be separated or combined, and the number of steps determines the adhesive classification [17,40,42,46].

## 3. Degradation at the Resin-Dentin Interface

**Figure 3:**
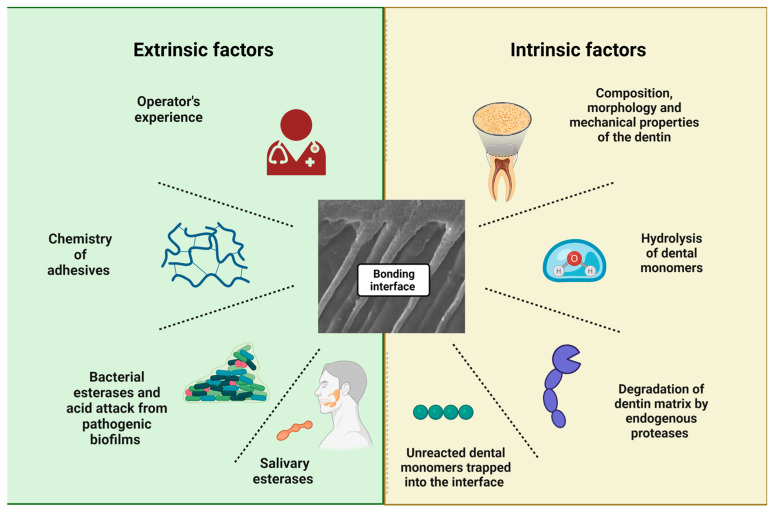
A summary of influencing factors on the degradation at the resin-dentin interface.

The degradation at the bonding interface is briefly defined as the disorganization and solubilization of collagen fibrils, associated with the hydrolysis and leaching of the adhesive resin from the interfibrillar spaces [47,48]. Collagen degradation was first described by Dayan et al. [49], then further explained by Tjäderhane et al. [50] and lastly rationalized by Pashley et al. [9]. The last group demonstrated that collagen degradation occurs over time in aseptic settings caused by intrinsic matrix proteases [9]. On the other hand, hydrolytic degradation occurs with the breakdown of the covalent bonds between polymers in the presence of water. It is also considered the prime reason for hybrid layer degradation [2,5,30]. Dentin is a hydrophilic substrate; it thus requires a hydrophilic resin monomer to infiltrate within the wet and demineralized dentin, causing the hybridization of the adhesive [51,52]. However, these hydrophilic monomers cause high water sorption, permitting water movement through the bonded interface and producing large water-filled channels that rapidly degrade the hydrophilic phase of adhesive [53]. Furthermore, water presence in the adhesive’s hydrophilic domains leads to the leaching of the solubilized resin, which increases the surface area allowing more water and soluble salivary enzymes to penetrate and cause bond hydrolysis [54]. Literature has summarized the factors that cause or accelerate the interface degradation into extrinsic and intrinsic factors, as summarized in Figure 3.

## 4. Extrinsic Factors Affecting Dental Bonding Longevity

The operator’s technique plays a huge role in achieving adequate resin-dentin adhesion. This can be summarized as the proper field isolation, following the exact recommendation of etching and bonding, the layering protocol followed in restoration application and the finishing/polishing procedures [55]. Furthermore, dentists’ knowledge and skills also influence the longevity of the treatments. The distinct ability of operators to decide between repairments and replacements of existing restorations plays a factor. The diagnostic methods during practice are different, as well as the decision-making [1].

In this context, it is clear that the longevity of dental restorations is closely associated with the replacement rate. Unfortunately, dentists are more prone to intervene in restorations made previously. As a result, patients who frequently change their dentists are more likely to get restorations replaced and, therefore, less longevity over time [1]. In addition to the techniques applied, the chosen material is essential for restoration success. With the different generations available, it is imperative to understand the chemistry of each one. For example, acetone-based adhesive systems show a higher annual failure rate than water-based adhesive systems. The rationale is that acetone-based systems require “wet bonding with a relatively small window of opportunity to achieve optimal hybridization, resulting in higher technique sensitivity” [54]. At the same time, the last published meta-analysis that compared acetone and alcohol-based adhesive systems showed no differences between these two systems in the clinical performance and survival rates in composite restorations placed in noncarious cervical lesions [56].

The choice of adhesive system applied and the respective clinical longevity has been extensively discussed in the literature. In 2014, a systematic review evaluated papers published in Medline and IADR abstracts in which adhesive systems were used in randomized clinical trials in non-carious cervical lesions from 1950 to 2013 [54]. The authors divided the adhesive systems into 3-step E&R, 2-step E&R, 2-step SE and 1-step SE. Self-etching agents were further divided according to pH into mild and moderately strong, with pH ≥ 1.5; and strong, with pH < 1.5.

The lowest annual failure rate (AFR) was observed for the 2-step SE adhesive system with moderate pH [2.5 (±1.5)], followed by the 3-step E&R adhesive system [3.1 (±2)] and the 1-step SE adhesive system with moderate pH [3.6 (±4.3)], with no statistically significant difference between these systems. Higher AFR was observed for strong 1-step SE adhesive systems [5.4 (±4.8)], 3-step E&R adhesive systems [5.8 (±4.9)] and strong 2-step SE adhesives [8.4 (±7.9)], with no statistical difference between these three adhesive systems. Selective acid etching on enamel did not influence the retention rate of self-etching adhesive systems. However, the authors noted that these results refer to the retention rate. If other parameters, such as marginal integrity, were evaluated, several studies have already shown the importance of selective acid etching in enamel [54].

Regarding the universal adhesive systems, follow-up time is short compared to other systems. However, suitable results have been observed so far for dentin adhesion. The most extended follow-up in a randomized clinical trial was in 2020 [57]. This analyzed the restorations’ survival in non-carious cervical lesions using universal adhesive and different acid etching strategies. After five years, the retention/fracture rates were: 93% (95% CI 81.4–97.6%) for the universal adhesive used in the E&R strategy; 93% (95% CI 81.4–97.6%) when used in the SE strategy; 88.4% (95% CI 75.5–94.9%) when the universal adhesive was used in the SE strategy with selective acid etching; and 81.4% (95% CI 67.4–90.3%) when used in the SE strategy without selective acid etching on enamel. The clinical behavior of the universal adhesive in the E&R strategy was superior to the SE, and enamel selective acid etching was strongly recommended when the SE strategy was used. Furthermore, some in-vitro studies suggested that adding a hydrophobic adhesive layer on dental tissues stabilizes the adhesive interface when the universal adhesive systems were used [55].

## 5. Intrinsic Factors Affecting Dental Bonding Longevity

Several influencing factors are implicated in the failure of the dentin-adhesive interface. Some are related to demineralizing dental hard tissues, while others are linked to dentin or resin degradation. The main ones responsible for restoration failure over time are pH fluctuations, humidity, temperature changes, aging, loading and fatigue.

The oral environment’s pH decrease can result from biofilm metabolism, an acidic diet or patient reflux. However, the most prevalent demineralization of enamel and dentin occurs when dental plaque (biofilm) is attached to the surfaces of these tissues. The oral cavity has more than 700 detected bacterial species [58], particularly 10^8^ to 10^10^ bacteria per millimeter found in saliva. Some can adhere to the tooth surface, initiating and forming a dental biofilm of microorganisms aggregation immersed in a sticky matrix [59,60]. Cariogenic bacteria that comprise part of this biofilm generate lactic acid as a product of dietary carbohydrates fermentation. Some factors related to the patient, such as the type of diet, salivary flow and oral hygiene, favor biofilm accumulation, and more nutrients are available to these microorganisms. The acidic environment favors maintaining and growing the acidogenic and aciduric bacteria population. When the pH level decreases to 5.5 or below, lactic acid dissolves the hydroxyapatite (HAp, Ca_10_(PO_4_)_6_(OH)_2_.

Concurrently, an acidic oral environment can lead to the hydrolysis of dental polymers. Particular concern refers to methacrylate groups due to the presence of esters, a chemical group susceptible to hydrolysis. Although the natural humidity of the oral environment would already lead to the hydrolysis of these materials, this fact is even worse in the presence of acids and enzymes from saliva or microorganisms, which catalyze hydrolysis processes.

From the perspective of the other side of the interface, dentin also degrades under the influence of water and enzymes, which may impact the degradation rates. In addition, in dentin, host-derived dentin proteases such as matrix metalloproteinases (MMPs) and cysteine cathepsins (CC) can promote extracellular matrix protein degradation, contributing to the failure of adhesion over time.

Matrix metalloproteinases (MMPs) are zinc- and calcium-dependent and originate from endogenous proteases secreted by odontoblast cells during dentin formation and mineralization [61,62]. MMP-2 and -9 are the most widespread in human dentin, in addition to MMP-3, -8 and -20 [63]. Dentin MMPs are trapped within hard tissues during mineralization and activated by physical and chemical conditions [64]. First, acids produced by cariogenic bacteria and acid etchants from the adhesive systems are the most common activators [65,66], followed by heat treatment and mechanical stress [65,66]. Next, MMP precursors are secreted as zymogens or pro-enzymes. These enzymes have a collagenolytic activity. This action is particularly undesired on the unhybridized collagen of hybrid layers and zones below, where the resin infiltration was incomplete during the operative steps.

After the activation of these endopeptidases, we may summarize the degradation of collagen in the following steps: (1) MMPs will hydrolyze the telopeptides of surface collagen dentin fibrils; (2) this event eliminates the globular telopeptides from collagen and creates spaces (previously masked) for other MMPs; and (3) other MMPs, classified as collagenases, bind to the sites to unwind the collagen molecule, leading to hydrolysis [67,68].

Cysteine cathepsin (CC) is another group of protease enzymes that hydrolyze dentin collagen, usually found in healthy and carious dentin [67]. Nascimento et al. [69] found a synergic effect between MMPs and cysteine cathepsin. Moreover, their activity was ten times higher in carious dentin than healthy ones.

Salivary esterases are also common components in the oral environment. For example, cholesterol esterase (CE) and Pseudocholinesterase (PCE) can hydrolyze the ester bond in the resin matrix [70,71], leading to adverse effects on the resin’s mechanical properties [72,73,74]. CE is also found in normal and inflamed gingiva, where monocyte macrophages produce it and increases with the non-specific immune response [70,75]. PCE is a form of glycoprotein, and has a low specificity for acetylcholine. It is found in various tissues [76]. Studies showed that both CE and PCE could degrade Bis-GMA, HEMA and TEGDMA in resins [77].

Neutrophils esterase are immune system component in the gingival crevicular fluid [78]. According to Gitalis et al. [79], neutrophils esterase can also degrade the bonding interface directly by promoting the release of 2,2-Bis [4(2,3-hydrohypropoxy)phenyl]propane (bisHPPP) from Bis-GMA degradation. In addition, neutrophils discharge myeloperoxidase and hypochlorite, which break ether bonds and catalyze the hydrolysis of ester bonds of resins [80].

Moreover, cariogenic biofilms can also produce proteolytic enzymes, contributing to the biodegradation of organic contents in the interface (collagen of dentin and polymer of dental materials) [81]. In the next section, we explored this topic.

## 6. Perspectives on Resin and Collagen Degradation: How Is Everything Connected?

While enamel is mainly inorganically composed, dentin is 55 vol.% mineral, 30 vol.% organic content (primarily type I collagen) and 15 vol.% fluids. Therefore, adhesive systems must show some hydrophilicity to infiltrate the dentin properly. In addition, these materials naturally present an ionic and hydrophilic character to improve dentin compatibility and make them suitable to interact with dentin’s complex and heterogeneous composition. Nonetheless, these features also make them prone to degradation and directly affect dentin permeability to oral fluids, making this tissue also likely to degrade.

The water sorption that dental adhesives suffer from leads to polymer chains breaking and a cascade of events that increasingly boosts the degradation processes of the polymer and the dentin collagen. Here, even the leaching compounds from the proper adhesive system will interfere with microbial growth at the interface and increase water sorption.

For instance, as aforementioned, acids and enzymes produced by biofilms can lead to the hydrolysis of monomers, increasing the degradation of polymers. The products of this degradation, whether non-polymerized monomers partially trapped in the polymeric network or by-products of molecular hydrolysis, are leached into the environment, potentially regulating biofilm virulence and increasing its development. In 2013, Bourbia et al. studied the hypothesis that *S. mutans* could produce esterase capable of degrading composite resin and adhesives. In this previous study, samples of restorative materials were prepared and incubated with *S. mutans* or culture medium without the bacteria for up to 30 days. In addition to evaluating the activity of bacterial enzymes, the authors identified a degradation product of BisGMA, showing that the longer the contact time with the bacteria, the greater the release of this product.

Furthermore, scanning electron microscopy confirmed the increased surface degradation of all materials with *S. mutans* compared to the medium without bacteria. The authors suggested that *S. mutans* indeed have esterase activities at levels capable of chemically degrading resin composites and adhesives, and that the degree of degradation depends on the chemical formulation of the material [82]. As another example, extensive research on *S. mutans* found that its presence enhances the surface roughness of resin composites, increasing bacterial adhesion on dental-bonding interfaces and triggering a snowball effect of biofilm accumulation and interface degradation [81].

Over time, the leaching of degradation products, unincorporated monomers and photoinitiators could alter bacterial growth and gene expression. This change probably occurs due to alterations in the gene expression of microorganisms. In 2018, Huang et al. evidenced that the SMU_118c gene, which is responsible for one of the main esterases produced by *S. mutans*, was achieved by degradation products from resins. Products such as BisHPPP, which comes from the degradation of BisGMA, increase the expression of these genes. So, in addition to pH and temperature, for example, these monomer hydrolysis products induce adaptation mechanisms in microorganisms by stimulating biofilm growth and increasing the regulation of genes essential for these bacteria’s virulence. The authors have suggested a series of events triggered by bisHPPP, a hydrophobic and weakly electrically charged molecule. In an acidic environment, bisHPPP accumulates around bacterial cells, disturbing the flow of fluids and nutrients. Therefore, these genes’ increased expression is associated with esterase production. The esterase catalyzes the monomers’ ester bonds, favoring the polymeric degradation process and potentially accelerating the interface’s biodegradation [83].

**Figure 4:**
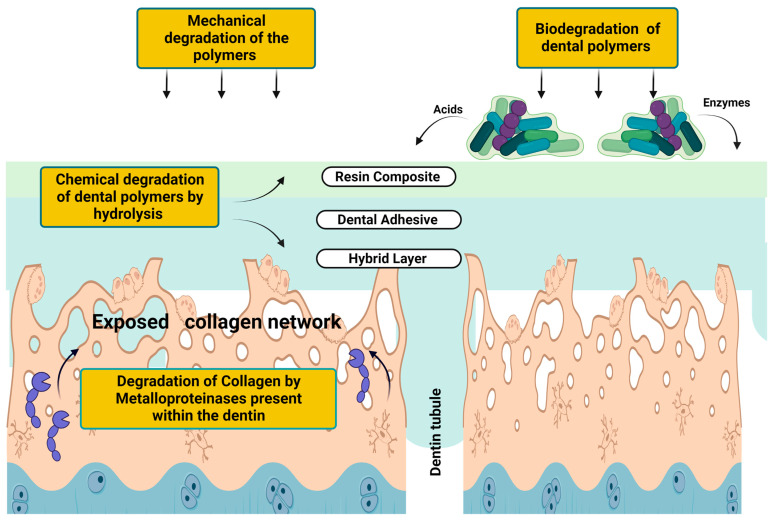
Schematic drawing illustrates the influencing factors contributing most to resin and collagen degradation phenomena.

Unfortunately, resins’ chemical degradation is concurrent with collagen’s chemical degradation. As previously mentioned, contact with phosphoric acid from E&R adhesive systems or acid monomers from SE adhesive systems will activate MMPs such as −2, −3, −8, −9 and −20 and cysteine cathepsins. In 2004, Pashley et al. revealed that the demineralized dentin suffered degradation without bacteria. This phenomenon occurs due to these active enzymes [9]. A summary of resin and collagen degradation is illustrated in Figure 4.

## 7. Current and Future Approaches to Reduce Degradation and Improve the Dental Bonding Longevity

The most explored anti-degradation strategy to date relies on developing dental monomers that are more hydrophobic or less susceptible to hydrolysis. For example, the monomer 2-hydroxyethyl methacrylate (HEMA) is a common dental monomer. HEMA is present in several adhesive formulations to improve surface wetting and prevent phase separation. Yet, HEMA promotes water sorption and hydrolysis at the adhesive interface, affecting long-term bonding to dentin. In this context, in recent years, acrylamides and methacrylamides have been suggested as potential candidates to replace HEMA. The amide group in acrylamides is more stable and resistant to hydrolytic degradation. Several acrylamide analogs of HEMA have been synthesized and investigated in the composition of newly formulated adhesives and resin composites. These monomers are ester-free, which decreases their chance of suffering hydrolysis [84]. For example, acrylamide cross-linker monomer N,N′-di acryloyl-4,7,10-trioxa-1,13-tridecanediamine, termed ‘FAM-201’ (Fujifilm, Tokyo, Japan), was revealed to be a promising candidate. This newly synthesized monomer showed significantly higher μTBS than HEMA over time (immediately and after six months of bond strength assessment) when applied in E&R and SE bonding modes.

Other alternatives are based on dentin treatments that reduce the chances of MMPs and CC binding to dentin. Chlorhexidine digluconate is the most-studied material for this purpose [85]. This molecule is usually employed in an aqueous solution after etching or before the adhesive placement. Then, it will electrostatically bind to dentin and protect the collagen fibrils against MMPs activity. However, further investigations are needed to assess longevity and durability. Other attempts are to chemically graft chlorhexidine into monomers and use them in adhesive formulation to increase its effect in dentin [85].

Quaternary ammonium compounds (QACs), such as benzalkonium chloride (BAC) and 12-methacryloyloxydodecylpyridinium bromide (MDPB), have also shown the ability to inhibit the enzymatic activity of MMPs through a mechanism similar to CHX by using its cationic charge. QACs have been used directly in dentin or incorporated into dental resins, promoting antibacterial activity besides the MMPs inhibition [86]. Inorganic materials, such as zinc oxide and copper, also decrease these enzymes’ effect [87].

Furthermore, quite a few investigations have explored the development of cross-linker compounds. These compounds have been tested to increase collagen cross-linking, preventing the loosening of collagen molecules. The cross-linkers intend to produce additional interfibrillar and intrafibrillar collagen cross-linked structures. The increased cross-link can improve the strength and stiffness of dentin by preventing collagen collapse [88,89]. Furthermore, the long-term application of cross-linkers showed protease inhibition through irreversible changes in dentin structures [90]. Examples of synthetic cross-linkers are aldehydes and carbodiimides, which are less toxic alternatives. Natural cross-linkers are riboflavins and proanthocyanidins [91]. Finally, plant-derived molecules, such as proanthocyanidins, curcumin and polyphenolic compounds, have been deeply explored due to their lower cytotoxicity [91,92,93]. In summary, the mechanism for all these strategies is based on creating covalent bonds between the amino groups of collagen proteins and the cross-linking agents. Table 2 displays the anti-degradation bonding strategies, target area and main mechanisms.

Some techniques have reported using several agents that increase dentins’ hydrophobicity. For example, replacing water rinsing clinically with ethanol in the E&R technique seems to reduce residual water in adhesive-dentin bonds, preventing collagen cleavage by matrix proteases [94]. Another example is the recent use of amphiphilic peptides. These molecules bind its hydrophilic end to dentin and expose the more hydrophobic side, increasing the hybrid layer’s hydrophobicity and resistance to hydrolysis [95]. Researchers have also been trying to evaluate the theoretical models to develop an ideal design of antimicrobial peptides to protect the resin-dentin interface. In addition to their antimicrobial properties, these peptides can mediate tooth remineralization depending on their chemical composition [99].

The use of self-etch adhesive systems should also be mentioned as a fundamental approach to reducing water from hybrid layers, especially those containing 10-methacryloyloxydecanethylene phosphoric acid (10-MDP). Since there is no clinical step of dentin water rinsing and the dentin thickness is not packed by monomers in the bottom of the hybrid layer, these adhesives are prone to show lower degradation over time. Furthermore, 10-MDP creates a stable salt after reacting with the HAp that surrounds collagen. Therefore, the collagen is not denuded and unprotected in the hybrid layer as occurs after using E&R adhesives systems, which removes the HAp around the fibers, releasing ions for the environment. There is no evidence of superiority between 3-step E&R adhesive systems and 2-step SE adhesive systems [54]. Both present a hydrophobic layer (adhesive layer) that assists in protecting the hybrid layer and dentin against degradation. However, it is worth mentioning that over time, more evidence will accumulate and the trend will be for 2-step SE adhesives proving superior behavior compared to 3-step E&R systems.

Another approach is the use of calcium-chelation dry bonding. The main idea is to use acids with different molecular sizes to remove the HAp between the fibers, not including those minerals which surround the fibers. Larger acidic molecules, such as polymeric chelators with different molecular weights, were recently tested to remove minerals from extra fibrillar dentin selectively. Compared to phosphoric acid, these molecules showed advantages in preserving the dynamic mechanical properties of dentin [97]. The effects of sodium carboxymethyl cellulose-based extra fibrillar demineralization conditioner were also analyzed. The authors evidenced that its application on dentin could improve bonding durability due to the inhibition of MMPs and the selective removal of minerals from extra fibrillar dentin [96].

Finally, there are attempts to induce the biomimetic remineralization of collagen using bioactive fillers. During demineralization, HAp is removed from the collagen and replaced by water. Then, the exposed fibers are vulnerable to degradation. Bioactive materials are used to “backfill the water-filled voids with nanometer-sized apatite crystallites during biomimetic remineralization.” Particles such as calcium phosphates and silicates have been widely explored to provide ions for the mineral’s formation within the fibers, restoring their mechanical properties [98].

## 8. Conclusions

The degradation of the dentin-adhesive interface, and consequently, the loss of seal at the tooth/resin, is believed to be the leading reason for premature failure of bonded dental restorations. This has resulted from a combinatorial effect of various mechanical and chemical factors, including the hydrolysis and enzymatic degradation of the exposed collagen and the adhesive resin.

In the past decade, the mechanisms of dentin/adhesive interface degradation and potential strategies to reduce or eliminate its progress have received much attention. It has led to many examples of highly promising pathways to better longevity of bonded dental restorations. Some approaches are especially promising, as they allow a more stable and resistant bonding interface to form, providing a platform to make bonded restorations more durable inside the mouth. Overall, the anti-degradation strategies are promising approaches for creating next-generation dental adhesives. However, the field is still developing in many ways, and much more exciting work on these versatile approaches is expected shortly.

In future research on improved dental adhesives and their bonding performance, we stress that it is imperative to work with standardized testing conditions that allow direct comparison of different studies. Moreover, the bonding performance should be reported over time to facilitate a better understanding of the degrading effects over modified and new adhesive strategies.

## Figures and Tables

**Table 1:** Role of dentin bonding system components.

Component	Action in E&R	Action in SE
Etchant	Dissolves the hydroxyapatite crystals of peritubular and intertubular dentin to open the tubules	Modifies the smear layer
Primer (Hydrophilic)	Produces a chemical bond between the primer’s carboxyl or phosphate group and collagen	Produces a chemical bond between the primer’s carboxyl or phosphate group of hydroxyapatite crystals covering the collagen
Bonding agent (Hydrophobic)	After polymerization, produces a hybrid layer through attachment to collagen fibrils and hydroxyapatite crystals	After polymerization, produces a hybrid layer through attachment to hydroxyapatite crystals

E&R: etch and rinse. SE: self-etch.

**Table 2:** Summary of the anti-degradation bonding strategies.

Anti-Degradation Strategy	Target Area	Mechanism	Ref.
Chlorhexidine digluconate	Inhibition of MMPs and CC	Bind to dentin and protect the collagen fibrils against MMPs activity	[85]
Development of ester-free monomers	Resistance to hydrolytic degradation	Increase the monomer hydrophobicity to be less susceptible to hydrolysis	[84]
Quaternary ammonium compounds (QACs)	Inhibition of MMPs and CC Antibacterial activity	Prevent MMPs and CC activity in dentin collagen Penetrates bacterial cell membrane causes cell death	[86]
Synthesis of collagen cross-linkers	Preservation of interfibrillar and intrafibrillar dentin	Prevent collagen collapse by improving dentin strength and stiffness through covalent bonds between collagen amino groups and cross-linking agents	[88,91]
Ethanol-wet technique	Increase dentin hydrophilicity	Replacing water rinsing by ethanol to prevent collagen cleavage	[94]
Amphiphilic peptide	Resistance to hydrolytic degradation	Increase hybrid layer’s hydrophobicity and resistance to hydrolysis	[95]
Self-etching technique	Dentin hybrid layer	Minimize the water presence in the hybrid layer and prevent demineralization	[54]
Calcium-chelation dry bonding	Removes calcium ions from dentin	Enhance penetration and adhesion of the bonding agent	[96,97]
Bioactive fillers for biomimetic remineralization	Remineralization of collagen fibers	Promote mineral deposition within collagen fibers	[98]

MMPs: matrix metalloproteinases. CC: Cysteine cathepsin.

## Data Availability

Not applicable.
